# Electrochemical Sensors with Carbon-Based Thick-Film Working Electrodes: Correlating Structure with Electrochemical Performance, Reproducibility, and Stability

**DOI:** 10.3390/s26165260

**Published:** 2026-08-19

**Authors:** Barbara Repič, Gregor Marolt, Andreja Benčan Golob, Goran Dražić, Danjela Kuscer

**Affiliations:** 1 Electronic Ceramics Department, Jožef Stefan Institute, Jamova cesta 39, 1000 Ljubljana, Slovenia; barbara.repic@ijs.si (B.R.); andreja.bencan@ijs.si (A.B.G.); goran.drazic@ijs.si (G.D.); 2 Jožef Stefan International Postgraduate School, Jamova cesta 39, 1000 Ljubljana, Slovenia; 3 Faculty of Chemistry and chemical technology, University of Ljubljana, Večna pot 113, 1000 Ljubljana, Slovenia; gregor.marolt@fkkt.uni-lj.si

**Keywords:** graphite, glassy carbon, carbon black, screen-printed electrode, integrated electrochemical sensor, reproducibility, stability

## Abstract

**Highlights:**

**What are the main findings?**

- Graphite thick-film electrodes with ordered sp^2^-hybridised layers exhibit higher conductivity and faster electron transfer than glassy carbon and carbon black with more localised structural ordering.
- Graphite-, glassy carbon-, and carbon black-based sensors exhibit reproducible behaviour (RSD < 3.5%), a potential window from −1.6 V to +1.0 V, and stable performance (>95% of the initial response) over 1000 CV cycles, with shelf stability > 1 month (RSD < 2%).

**What are the implications of the main findings?**

- The morphological and structural properties of carbon materials dictate their electrochemical behaviour and thus sensor performance.
- Reproducible and stable behaviour of integrated sensors enables reliable electroanalysis of target analytes.

**Abstract:**

Integrated electrochemical sensors (IESs) offer rapid and efficient detection of environmental pollutants, but their broader practical implementation requires overcoming common challenges associated with reproducible fabrication and long-term stability. In this work, these challenges were addressed using a thick-film approach to fabricate IESs with graphite-glass, glassy carbon, and carbon black working electrodes (WEs) by screen printing followed by firing at 850 °C. The relationship between the structure of the carbon-based WEs and the electrochemical performance of the IESs was systematically investigated using cyclic voltammetry (CV) in combination with X-ray powder diffraction, transmission electron microscopy (TEM), and scanning TEM. The analyses revealed distinct morphologies and structural ordering of the carbon WEs, which directly affect their electron-transfer kinetics, adsorption behaviour, capacitive response, and electrochemically active surface area. The ordered structure of the graphite-glass WE was associated with lower capacitance and faster electron-transfer kinetics, as determined from the CV response of the IES. In contrast, the disordered structure of the carbon black WE was associated with higher capacitance and slower kinetics of the IES. The glassy carbon-based IES exhibited kinetics similar to those of the carbon black-based IES, but with the lowest capacitance, resulting in the greatest signal definition. Consequently, although all IESs exhibited wide operating potential windows (−1.6 V to +1.0 V vs Ag/AgCl) and fast heterogeneous electron-transfer kinetics towards the [Fe(CN)_6_]^3−/4−^ redox probe (7.6 × 10^−3^–15.5 × 10^−3^ cm s^−1^), the carbon materials differed in their electrochemical response and signal definition. Importantly, all IESs demonstrated excellent reproducibility (relative standard deviation < 2.4%), operational stability with less than 5% signal loss after 1000 CV cycles, and shelf-life stability exceeding 30 days. These findings demonstrate that tailoring the carbon structure of the screen-printed thick-film WEs enables reproducible fabrication of stable and reliable IESs while providing a versatile strategy for tuning their electrochemical performance towards application-specific requirements.

## 1. Introduction

Electrochemical sensing is widely used in environmental monitoring, food safety, biosensing, medical diagnostics, forensics, and industrial process control [1,2,3,4]. Recent advances in electronics and manufacturing have driven the miniaturisation of electrochemical systems and the development of integrated electrochemical sensors (IESs), such as screen-printed electrodes (SPEs), in which all components are integrated onto a single substrate [3,4]. Thick-film technologies enable reproducible, scalable, and cost-effective fabrication of IESs, making them particularly attractive as disposable devices. The performance and practical implementation of IESs depend strongly on the WE material and its structural properties, highlighting the need for fabrication strategies that ensure reproducibility and long-term stability [2,5,6].

Carbon-based materials are widely employed as working electrodes (WEs) owing to their high electrical conductivity, wide electrochemical potential window, low background current, chemical stability, biocompatibility, and low cost [6,7,8]. While glassy carbon is widely used for classical disc electrodes, its application in thick-film form remains less explored. Our previous study [9,10,11] on thick films prepared from carbon materials with different particle sizes showed the applicability of graphite (G) as a commonly used material in SPEs and glassy carbon (GC) as an analogue of bulk electrodes, while carbon black (CB) was demonstrated as a material with a high surface area for potential adsorption-based sensing. Differences in electrochemical performance were observed and attributed to post-deposition treatment, surface morphology, structure, and electrical properties. These earlier studies primarily focused on thick-film processing, structural characterisation and fundamental electrochemical behaviour. However, systematic evaluation of processing reproducibility, operational durability, and shelf-life stability of IESs based on these thick-film materials has not yet been reported.

Although G, GC, and CB all consist predominantly of sp^2^-hybridised carbon, they differ substantially in their degree of structural order [7,12]. G is characterised by a high degree of structural ordering and consists of crystallites arranged in stacked graphene layers with long-range three-dimensional order. In contrast, GC is a non-graphitising carbon composed of interconnected graphene fragments with small crystallites, larger interlayer spacing, and no long-range stacking order. CB consists of aggregates of nanometre-sized curved graphitic shells with a highly disordered structure [7]. These structural differences influence the electronic properties of carbon materials, as well as their electrochemical performance via physicochemical characteristics of the electrode surface, including edge-plane and basal-plane exposure, defect density, surface functional groups, and surface oxides [7]. Although these materials have been shown to exhibit distinct electron-transfer kinetics, capacitive behaviour, and interactions with species in solution [7], the mechanisms underlying these differences remain unclear.

Besides the intrinsic properties of carbon materials, the sensor performance and reproducibility are also affected by the thick-film processing conditions [7]. Thick-film fabrication involves dispersing carbon particles in an organic vehicle, followed by deposition and curing [13]. Parameters such as paste formulation, the printing process and post-deposition curing conditions determine the structure, microstructure and surface properties of the resulting thick films [14,15]. We demonstrated that, during annealing, the organic phase from the screen-printing paste decomposes and affects the ordering of the graphene layers, the sheet resistance of the thick films and their electrochemical performance [10]. Characterisation and understanding of carbon thick-film structure are essential for interpreting and optimising its electrochemical performance.

Reliable performance of IESs therefore requires optimisation of both the carbon electrode material and the fabrication process. Thick-film electrodes based on G, GC and CB, annealed at 850 °C, exhibit distinct electrochemical behaviour arising from differences in their microstructure and surface morphology [9,11]. While thick-film processing, structural characterisation and fundamental electrochemical behaviour have been the primary focus of our previous studies [9], systematic evaluation of fabrication reproducibility, operational durability and shelf-life stability of IESs based on these materials has not yet been reported.

This study addresses a key requirement for the practical implementation of IESs by demonstrating a fabrication approach that yields reproducible and stable IES performance. The novelty of this work lies in the systematic comparison of three different carbon thick-film WE materials, namely G, GC, and CB, fabricated and electrochemically evaluated under identical conditions. Hexacyanoferrate (II/III) was selected as a well-established redox probe with a well-understood electron-transfer mechanism, enabling direct comparison of the intrinsic properties of the electrode materials while minimising analyte-specific effects. In contrast to our previous studies, which primarily addressed structural characteristics and basic electrochemical behaviour of the carbon thick-film WEs, this work establishes direct correlations between the nanoscale structure of the WEs and electrochemical performance, fabrication reproducibility, operational stability, and shelf-life of the IESs. By integrating these aspects within a single systematic study, this work provides new insights into the relationship among WE structure, IES performance, and IES stability and establishes practical guidelines for processing conditions and the selection of carbon materials for reliable, application-specific IESs.

## 2. Materials and Methods

### 2.1. Fabrication of Integrated Electrochemical Sensors

IESs consist of a thick-film, carbon-based WE with a diameter of 4.2 mm, an Ag/AgCl reference electrode (RE), and a Pt counter electrode (CE), as shown before [11]. All electrodes and electrical contacts were screen printed onto an alumina substrate (96% Al_2_O_3_, Rubalit 708S, CeramTec) using a screen printer (P-250AVF, KEKO Equipment, Slovenia). The electrical contacts and CE were prepared from a commercial platinum paste (9141 R, Du Pont), the RE from a commercial Ag/AgCl paste (60/40) (Sigma Aldrich), and the WEs from in-house screen-printing pastes made from three different powders, namely: (i) a mixture of 75 wt% graphite (graphite flake, 99.8%, Alfa Aesar) and 25 wt% glass powder (Steklarna Hrastnik, Slovenia), (ii) glassy carbon powder (spherical powder, ≤100%, Thermo Fisher), and (iii) carbon black powder (special black 250, Evonik Carbon Black) [9,10]. First, the Pt electrical contacts and CE were printed and fired at 1000 °C for 1 h in air, followed by printing and firing the carbon-based WEs at 850 °C for 30 min in argon to prevent carbon degradation in an oxygen-containing atmosphere [9,11], and then printing and drying the Ag/AgCl RE at 120 °C for 10 min in air. Lastly, the electrical contacts were manually covered with a nitrocellulose-based polymer, which was air-dried at room temperature, to obtain final IESs with graphite-glass (GG), GC, and CB WEs.

### 2.2. Physicochemical Characterisation of Integrated Electrochemical Sensors

The thickness (*t*) and root-mean-square surface roughness (*R*_q_) of all thick-film electrodes were measured using a contact stylus profilometer (DektakXT Advanced System, Bruker, USA) with the software Vision 64 (Bruker, USA). The sheet resistance (*R*_s_) of all thick-film electrodes was determined by a four-point probe technique using a source measure unit (Keithley 237, Keithley, USA) and a geometric correction factor of 0.795 The material resistivity (*ρ*) was calculated by multiplying *R*_s_ by the thickness of the films. Details are given in [9].

The contact angle of 100 mM phosphate buffer solution (PBS) on the GG, GC, and CB thick films was measured with a tensiometer (DSA20E, Krüss, Germany) at room temperature.

The X-ray diffraction (XRD) patterns of the GG, GC, and CB thick films were collected using an XRD diffractometer (Empyrean, Malvern PANalytical) with Cu Kα1 radiation (*λ* = 1.5406 Å) in grazing-incidence (GI) mode (GI-XRD), with the GI angle fixed at 0.2°. Data were acquired over the 2*θ* range from 20° to 60° with a step size of 0.02°. The phases were identified using X’Pert HighScore Plus 3.0e (PANalytical) and the PDF-5+/Web 2025 database.

Structural, morphological, and chemical investigations of the GG, GC, and CB thick films at the nanoscale were performed using (scanning) transmission electron microscopy ((S)TEM), selected-area electron diffraction (SAED), energy-dispersive X-ray spectroscopy (EDXS), and electron energy-loss spectroscopy (EELS) on a Jeol ARM 200CF microscope (Jeol Ltd., Japan) equipped with a Jeol Centurio EDX with a 100 mm^2^ SDD detector and a Gatan Quantum ER Dual EELS spectrometer (Gatan, USA). The analysed specimens were prepared by sandwiching the films between two alumina substrates, embedding them in epoxy resin, and subsequently thinning by mechanical grinding and dimpling, followed by final Ar-ion milling until perforation using a precision ion polishing system (PIPS, Gatan PIPS Model 691, USA).

### 2.3. Electrochemical Characterisation of Integrated Electrochemical Sensors

All chemical reagents used for electrochemical measurements, preparation of phosphate buffer solution (PBS), and equimolar hexacyanoferrate(II/III) (HCF) redox probe in PBS are described in [9]. Electrochemical analysis of the IESs was carried out using a potentiostat (Multi Autolab M 204, Metrohm, Netherlands) operated with Nova 2.1.5 software. Cyclic voltammetry (CV) scans were performed at 25 ± 0.5 °C in a 5 mL electrochemical cell in 100 mM PBS, pH 7, and in a model solution containing 5 mM HCF in 100 mM PBS. Before each voltammetric experiment, all solutions were purged with nitrogen for 5 min, and purging was maintained throughout the measurement. Prior to CV measurements, the uncompensated resistance (*R_u_*) was determined using a positive-feedback method for Ohmic iR-drop evaluation. All CVs were recorded first in blank PBS and then in HCF solution, all without *R*_u_ compensation.

The CVs in HCF and PBS on the IESs with GG, GC, and CB WEs, denoted GGES, GCES, and CBES, respectively, were recorded between −0.25 V and +0.45 V vs the Ag/AgCl RE. The potential window for the CV measurements in HCF was selected based on the open current potential (OCP) value measured in HCF solution of approximately 0.1 V. The potential limits were set 0.35 V more positive and negative relative to this OCP. Scans started at −0.25 V in the positive direction with a step potential of 2.44 mV at scan rates of 50, 75, 100, 150, and 200 mV s^−1^. The first set of measurements was performed in PBS by conducting five cycles at 100 mV s^−1^ to determine the capacitive current (*I*_cap_). The second set of measurements in PBS was performed by recording three cycles at the five selected scan rates to determine the double-layer capacitance (*C*_dl_) [16]. The same measurements were repeated in HCF, from which the cathodic and anodic peak potentials (*E*_pc_ and *E*_pa_, respectively) and peak currents (*I*_pc_ and *I*_pa_, respectively) were determined. These were used to calculate the peak-to-background (P/B) current ratio, the ratio of cathodic to anodic peak current (*I*_pc_/*I*_pa_), the peak-to-peak potential separation (Δ*E*_p_), and the formal potential (*E*_½_) as described previously [9]. From the CVs recorded in HCF at different scan rates, the electrochemically active surface area (*A*_ecsa_) was calculated using the modified Randles–Ševčík equation for a quasi-reversible reaction [17,18] and the standard heterogeneous electron-transfer rate constant (*k*^0^) using the Nicholson method [19,20]. The equations, parameter values, and detailed calculation procedures were reported in our previous work [9]. The diffusion coefficient of the oxidised, i.e., hexacyanoferrate (III) species, *D*_O_ = 7.26 × 10^−6^ cm^2^ s^−1^ was used for the calculations [21]. The actual surface area (*A*_real_) was calculated by dividing *A*_ecsa_ by the electrode’s geometric surface area (*A*_geo_ = 13.85 mm^2^ for *Φ* = 4.2 mm). The peak current density (*J*_p_) was calculated by dividing the average value of the absolute peak currents *I*_pc_ and *I*_pa_ by *A*_ecsa_.

Sensor reproducibility was assessed from the relative standard deviation (RSD) of *I*_pa_ and Δ*E*_p_ measured in HCF using three independently prepared sensors. Operational stability was evaluated by recording 1000 consecutive CVs in HCF with two independently prepared sensors and by monitoring changes in *I*_pa_ and Δ*E*_p_ relative to the first CV scan. Additionally, the Ag/AgCl pseudo-RE of the two independently prepared IESs was connected as the WE against a conventional Ag/AgCl/3 M KCl RE, and the open current potential (OCP) in HCF was measured for 1 h to test the stability of the pseudo-RE alone. Shelf-life stability was determined from measurements of four independently prepared sensors stored under ambient laboratory conditions for the specified storage period prior to electrochemical characterisation in HCF, with changes in *I*_pa_ and Δ*E*_p_ evaluated relative to freshly prepared IESs.

The operating potential window (OPW) of the IESs was determined based on a qualitative study of a series of CVs measured at a scan rate of 100 mV s^−1^ in PBS by incrementally changing the upper and lower potential limits by 50 mV increments as described previously in [11,22]. The cathodic and anodic limits of the OPW were identified as the onset of the hydrogen evolution reaction (HER) and carbon oxidation, respectively, at which the current exceeded *I*_pc_ and *I*_pa_ obtained for the HCF redox probe.

## 3. Results and Discussion

### 3.1. Physicochemical Characterisation of Carbon-Based Thick Films

**Figure 1:**
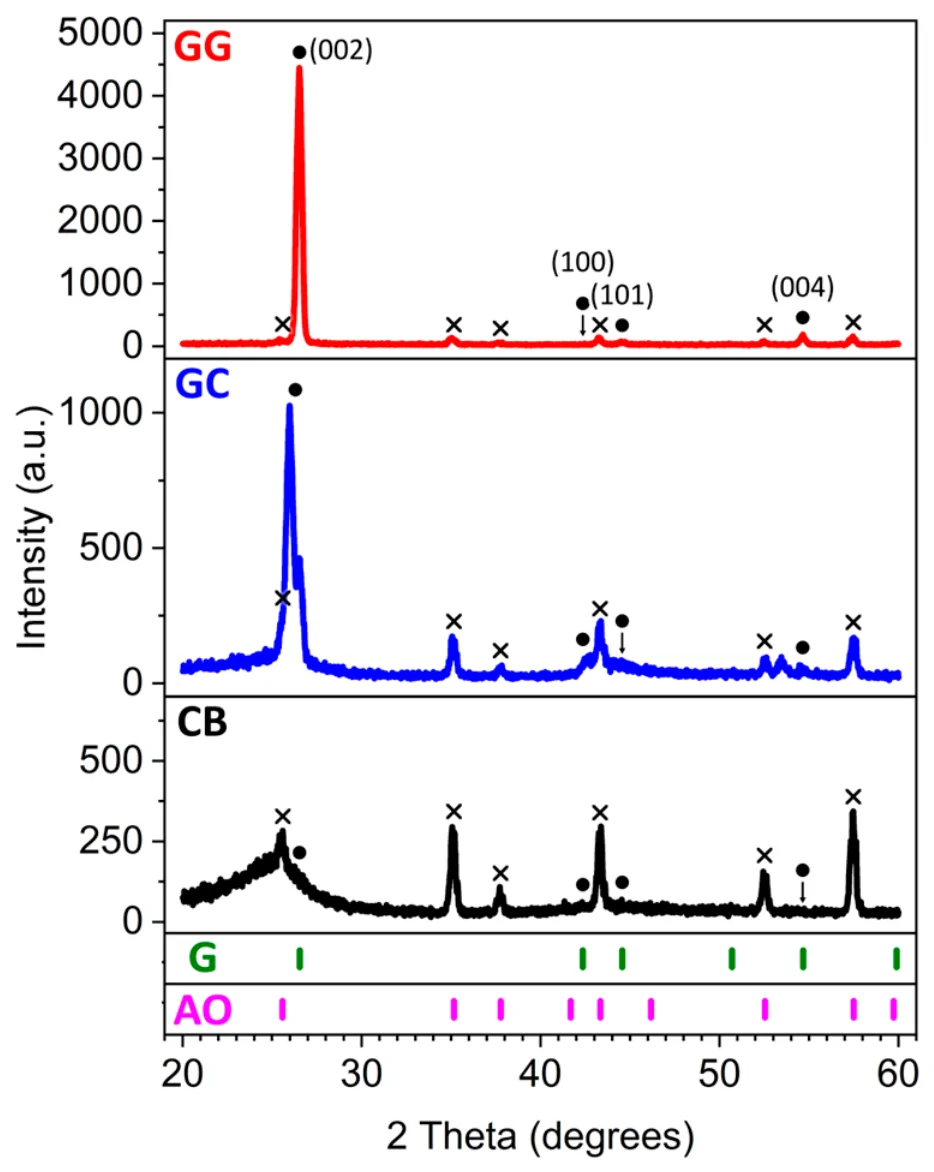
Grazing-incidence X-ray diffraction patterns of graphite-glass (GG, red), glassy carbon (GC, blue), and carbon black (CB, black) thick films on an alumina substrate, with the positions of the reference diffraction patterns of graphite (G, green) and alumina (AO, magenta) marked with ● and ✕, respectively.

**Figure 2:**
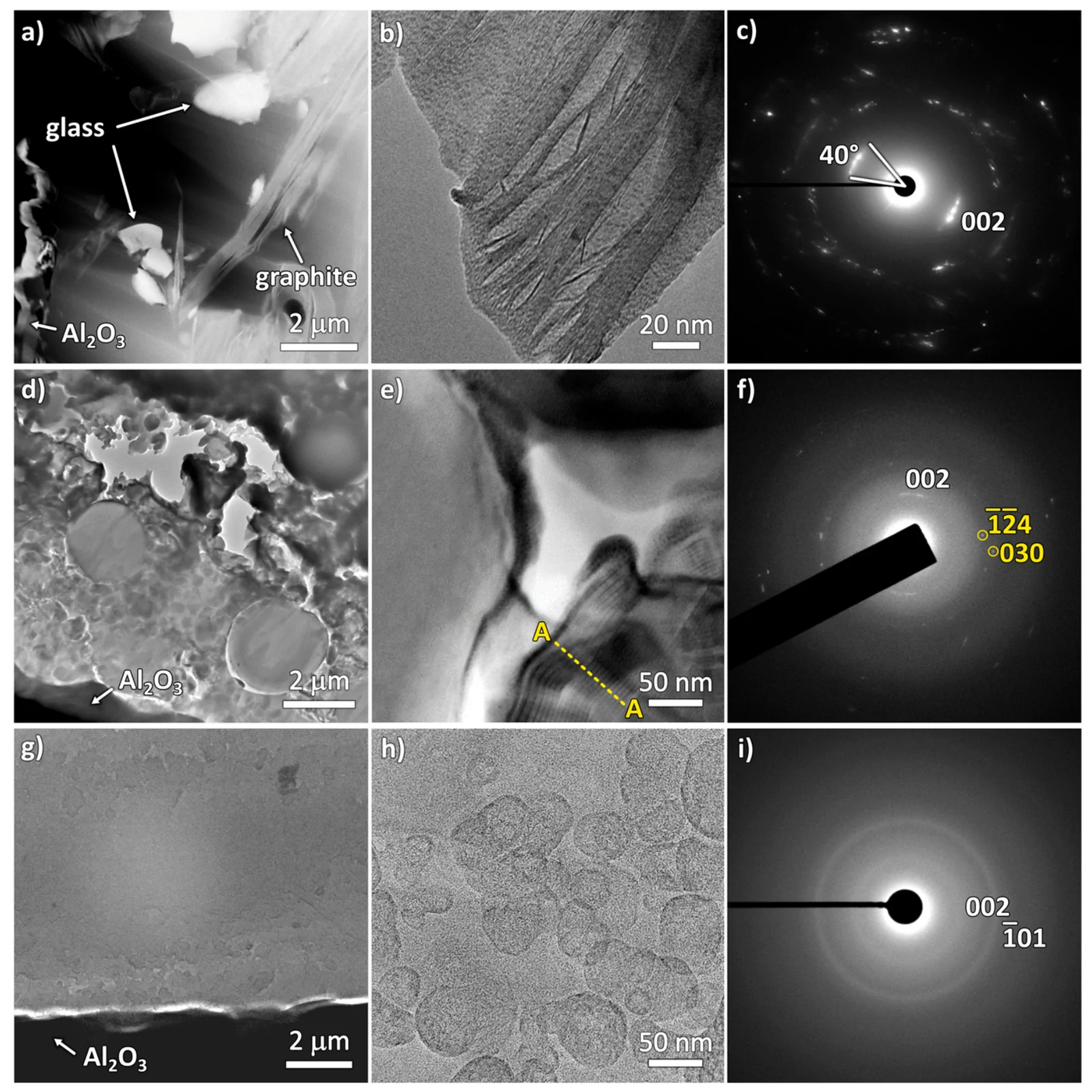
Scanning transmission electron microscopy images at different magnifications and selected-area electron diffraction patterns of graphite-glass (**a**–**c**), glassy carbon (**d**–**f**), and carbon black (**g**–**i**) thick films, showing indexed graphite arcs or diffuse rings (white) and alumina spots (yellow). Reflections with hexagonal indices are expressed in three-index (hkl) notation for consistency. The 40° angle of the diffraction arc marks the local misorientation of the (002) diffraction. Line A–A marks the area with partially periodic, distinct graphitic stacking.

The GI-XRD patterns and STEM/SAED data of the GG, GC, and CB thick films are presented in Figure 1 and Figure 2, respectively, while the EDXS and EELS spectra are shown in the Supplementary Information in Figure S1 and Figure S2, respectively. The diffraction patterns of all carbon films exhibit characteristic peaks corresponding to the hexagonal crystal structure of graphite (G, PDF 00-56-0159) and low-intensity reflections corresponding to corundum (AO, PDF 01-075-6775), originating from the substrate. Similar crystal structures have previously been reported for the powders [9], indicating that annealing does not significantly affect the structure or crystallinity of the carbon films. STEM images confirm that the morphology within the carbon films resembles the morphology at the film surface, which has been reported to depend strongly on the powder morphology [9]. The differences in structural and morphological properties are further discussed later in this section for each material separately.

#### 3.1.1. Graphite-Glass (GG) Thick Film

The GG film exhibits a diffraction pattern with characteristic, sharp peak reflections at 26.6° and 54.7°, assigned to the (002) and (004) basal planes, respectively, while the peaks at 42.4° and 44.5° originate from the (100) and (101) in-plane lattice reflections of graphitic structures. This indicates a high 3D long-range ordering typical of graphitic carbons [12]. No distinct diffraction features attributable to the added glass phase were observed.

The dark-field STEM (DF-STEM) image (Figure 2a) shows two morphologically different phases with distinct chemistry, confirmed by EDXS analysis (Figure S1). The irregular, micrometre-sized bright particles correspond to the SiO_2_-based glass phase, while the elongated grey regions correspond to the graphitic phase. At higher magnification (Figure 2b), the graphite phase exhibits a textured, anisotropic morphology, composed of elongated, highly aligned graphitic domains with a layered internal structure. The basal planes of these domains are aligned parallel to the long axis of the elongated features. The stacked graphitic layers exhibit a local misorientation on the order of ±20° within an amorphous carbon matrix. This is further supported by SAED, where arcs corresponding to the (002) planes are observed, along with two diffuse rings (Figure 2c).

The EELS spectra (Figure S2) further confirm that the more ordered, elongated layered features (GG-A) exhibit a distinct electronic structure and a higher degree of local bonding order compared to the adjacent regions (GG-B). The carbon K-edge spectra of GG-A exhibits sharp and well-defined characteristic π* (~285 eV) and σ* (~292 eV) peaks, along with additional σ* features appearing on the high-energy side of the σ* peak. The latter indicates extended periodicity and a high degree of symmetry in the atomic arrangement, consistent with a crystalline sp^2^-bonded carbon structure. In contrast, the GG-B regions exhibit a comparatively weaker π* peak, followed by a largely featureless spectral profile over the subsequent ~20 eV, consistent with a disordered, amorphous-like carbon structure. Since π* and σ* features are associated with sp^2^- and sp^3^-hybridised bonds, respectively [23,24], the ratio of the π* to σ* peak relates to the fraction of sp^2^-hybridised carbon atoms in the material [25,26]. Based on visual inspection, the amorphous regions have a lower fraction of sp^2^-hybridised carbon atoms than the elongated layered features. Overall, all methods confirm that the GG film consists predominantly of a highly ordered sp^2^ network, along with some amorphous carbon within the graphite phase and an additional glass phase.

#### 3.1.2. Glassy Carbon (GC) Thick Film

The GC film exhibits a diffraction pattern with lower intensity, with partially overlapping reflections at 25.9° and 26.5° of unequal intensity, assigned to the (002), and reflections at 53.4° and 54.7°, assigned to the (004) basal planes. Additionally, there are diffuse and overlapping reflections in the neighbourhood of the graphite (100) and (101) reflections between ~42° and ~46°. The splitting of the (002) and (004) reflections could be attributed to the coexistence of regions with different degrees of structural ordering. Similar phenomena in GC were observed by [27]. The contribution at lower diffraction angles corresponds to a larger interlayer spacing (d_002_ ~ 0.341 nm), characteristic of carbon with turbostratic stacking disorder, while the peak at higher angles is associated with more ordered graphitic stacking (d_002_ ~ 0.335 nm). Overlapping (00l) and (hk) reflections also indicate the presence of 2D long-range ordering within the hexagonal layers but a lack of crystallographic ordering in the third dimension, which is typical of non-graphitic carbons such as glassy carbon [12].

From the DF-STEM image (Figure 2d), micrometre-sized spherical particles surrounded by smaller sub-micrometre-sized particles are visible, in agreement with the morphology and particle size of the initial powder [9]. Reduced particle sphericity and the formation of interparticle necks are observed within the film. This partial interparticle coalescence indicates thermally induced bonding and structural consolidation during annealing. At higher magnification (Figure 2e), most regions do not exhibit distinct graphitic stacking, while in some areas (marked with the A–A line), there is partially periodic layered contrast with a period of 5–10 nm. These features are not sufficiently periodic to be assigned to the presence of different crystal structures at the atomic level but could be attributed to structural disorder or planar defects within the same graphitic framework. In SAED (Figure 2f), the GC film displays diffuse rings together with a weak diffraction arc and discrete diffraction spots. The arc is attributed to the (002) reflection of graphitic carbon, which together with the diffuse rings indicates the coexistence of locally ordered graphitic regions with disordered carbon. The discrete diffraction spots, on the other hand, indicate a higher degree of crystallinity compared to the graphitic contribution and are assigned to the Al_2_O_3_ substrate. This was further supported by comparison with simulated SAED patterns of alumina and graphite, which confirmed that the spots occur in regions where no graphite diffraction is expected and coincide with reflections of alumina. Although XRD suggests the presence of two overlapping contributions associated with regions of different stacking order and interlayer spacing, the difference between them is below the resolution of SAED, and the observed arc likely represents a combined contribution from both. The difference could also be attributed to the different sampling volumes of the techniques, as XRD provides an average structural response over a much larger volume, whereas SAED probes only a highly localised region.

The EELS spectrum (Figure S2) of the GC film exhibits both π* and σ* peaks, however, the less pronounced π* peak suggests largely disordered (amorphous-like) local bonding on the atomic scale. This is typical of commonly reported turbostratic structures with misoriented and distorted graphitic stacking. Also, less structured σ* features indicate a more disordered sp^2^ network. The smaller π* to σ* peak ratio also indicates a lower degree of graphitic ordering with a smaller fraction of sp^2^-hybridised carbon atoms. Overall, one can conclude that the GC film consists predominantly of disordered carbon with local graphitic ordering.

#### 3.1.3. Carbon Black (CB) Thick Film

The CB thick film exhibits a diffraction pattern with a relatively high background and two broad, low-intensity peaks at ~25° and ~43°, assigned to the (002) reflection and (10) band. These peaks are partially masked by reflections corresponding to the alumina substrate. The pattern of graphitised CB has been reported to resemble that of polycrystalline graphite with small crystallites [7]. The lower intensity of the (002) reflection due to the broadening effect from having a small particle size has also been previously reported for CB powder [9]. However, its position at a lower diffraction angle also suggests that the average distance between carbon layers (*d*_002_ ~ 0.355 nm) exceeds the typical interlayer spacing in graphitic and non-graphitic carbons [28]. The broad reflections indicate a very limited long-range structural order, characteristic of amorphous carbon such as CB [12].

The DF-STEM image (Figure 2g) shows a densely packed morphology, whereas at higher magnification (Figure 2h), individual particles with an average diameter of approximately 50 nm are visible. Concentric layered contrast within the particles suggests curved graphitic shells, characteristic of carbon nano-onions [29]. The SAED pattern (Figure 2i) shows homogeneous parallel rings, indicative of amorphous carbon, however, the pattern is in good agreement with the simulated SAED pattern of graphite crystallites several nanometres in size.

The EELS spectrum (Figure S2) of the CB film exhibits both π* and σ* peaks, with smeared σ* features at higher energy loss indicating highly disordered carbon. There is also an additional shoulder at ~287 eV, which is most likely associated with curved graphitic structures or fullerene-like bonding configurations [30], consistent with an onion-like carbon morphology. The smaller π* to σ* peak ratio is indicative of more disordered carbon with a smaller fraction of sp^2^-hybridised carbon atoms. We can conclude that the CB film consists of highly disordered carbon with some structural order within the carbon nanoparticles, which exhibit curved graphitic shells.

It has been demonstrated that the structural and morphological properties of the investigated carbon thick films resemble those reported for their corresponding powders [9]. All films exhibit a graphitic structure but with significant differences in the degree of ordering and locally disordered bonding. GG exhibits the highest degree of graphitic ordering and extended bonding periodicity, GC shows structural heterogeneity with mixed ordering and more disordered bonding, whereas CB has a predominantly disordered bonding environment with limited stacking order. These differences suggest variations in the bulk electronic and surface properties of carbon electrodes, which affect their electrochemical behaviour, including capacitive behaviour, electroactive surface area, electron-transfer kinetics, and adsorption properties.

Since electrochemistry is fundamentally based on interfacial phenomena, the nature of the carbon electrode surface is of obvious importance. Besides surface roughness, the structural differences observed by XRD, SAED, and EELS may also affect the surface composition, which dictates the surface reactivity, capacitance, and adsorption properties of these materials. The edges of graphitic layers and defects in basal planes are known to be highly reactive towards surface oxidation [7]. Therefore, more disordered carbon materials with a higher ratio of edge to basal planes are assumed to have greater surface oxide coverage. The surface oxides of graphite-glass film electrodes have previously been investigated by X-ray photoelectron spectroscopy (XPS) [11]. These electrodes were prepared under the same annealing conditions in an Ar atmosphere as reported in this work. The surface atomic O/C ratio of 0.7% for as-prepared electrodes indicates a low amount of surface oxides. Although surface oxidation of carbon thick films was not investigated in this work, differences in oxide coverage also affect the hydrophilicity of the film and, consequently, the contact angle of water at the carbon thick film surface. The contact angles of PBS electrolyte were 115° ± 1°, 107° ± 1°, and 61° ± 1° for GG, GC, and CB, respectively. CB exhibited the lowest contact angle, indicating the greatest surface wettability among the investigated materials. The higher hydrophilicity of CB is consistent with a higher degree of surface oxidation, as expected from its more disordered structure. The improved electrolyte wetting is expected to facilitate the formation of the electrochemical double layer over a larger accessible surface area and therefore contributes to the substantially higher double-layer capacitance (*C*_dl_). The presence of oxygenated functional groups is also expected to provide a greater tendency for adsorption of species from solution.

### 3.2. Physical Properties of Integrated Electrochemical Sensors

All electrodes of the investigated IESs adhered well to the alumina substrate and exhibited uniform layer thickness across their surfaces. The addition of glass in GG improves the adhesion of graphite to alumina [11], while GC and CB exhibit sufficient adhesion on their own. Representative thickness profiles of all IES electrodes are shown in Figure S3, and average values of the thickness (*t*), surface roughness (*R*_q_), sheet resistance (*R*_s_), and resistivity (*ρ*) of each electrode are summarised in Table 1.

The GG, GC, and CB thick films exhibit different thicknesses, surface roughnesses, and sheet resistances despite being processed under identical conditions. These variations can be attributed to differences in particle size of the GG, GC, and CB powders, which influence the resulting microstructure and consequently the sheet resistance of the thick films [9]. The GG thick films, made from a few micrometre-sized graphite and glass particles a few micrometres in size, exhibit the highest surface roughness, whereas the smaller particle size of GC and CB results in smoother thick-film surfaces. The GG thick film has lower sheet resistance than the GC and CB thick films. The addition of glass to graphite results in lower surface roughness compared with a pure G thick film but does not significantly affect the conductivity of GG. The higher intrinsic conductivity of GG has been attributed to a higher degree of π-electron delocalisation in a highly ordered structure, compared with GC and CB, where charge carriers are more localised due to greater disorder. The electrical conductivity of GG, GC, and CB in film form also depends on particle size and the microstructure of the thick film, which affect the charge-transport pathways [14,31,32,33]. Comparing the resistivities with reported values for randomly oriented graphite (Ultra Carbon UF-4S grade, Ultra Carbon Corporation) of 1 mΩ cm, GC treated at 2000 °C (GC-20, Tokai Carbon) of 4.2 mΩ cm, and CB (Spheron 6, Cabot Corporation) of 50 mΩ cm [7], it can be concluded that the resistivities of GG, GC, and CB thick-film electrodes are within the same range and more closely resemble the reported value for CB.

The Pt electrode had the lowest thickness of all the electrodes, a low surface roughness, and a very low sheet resistance. The Ag/AgCl electrode was thicker, had the highest surface roughness, and also a low sheet resistance.

### 3.3. Electrochemical Properties of Integrated Electrochemical Sensors

#### 3.3.1. Response to [Fe(CN)_6_]^3−/4−^ Redox Probe

**Figure 3:**
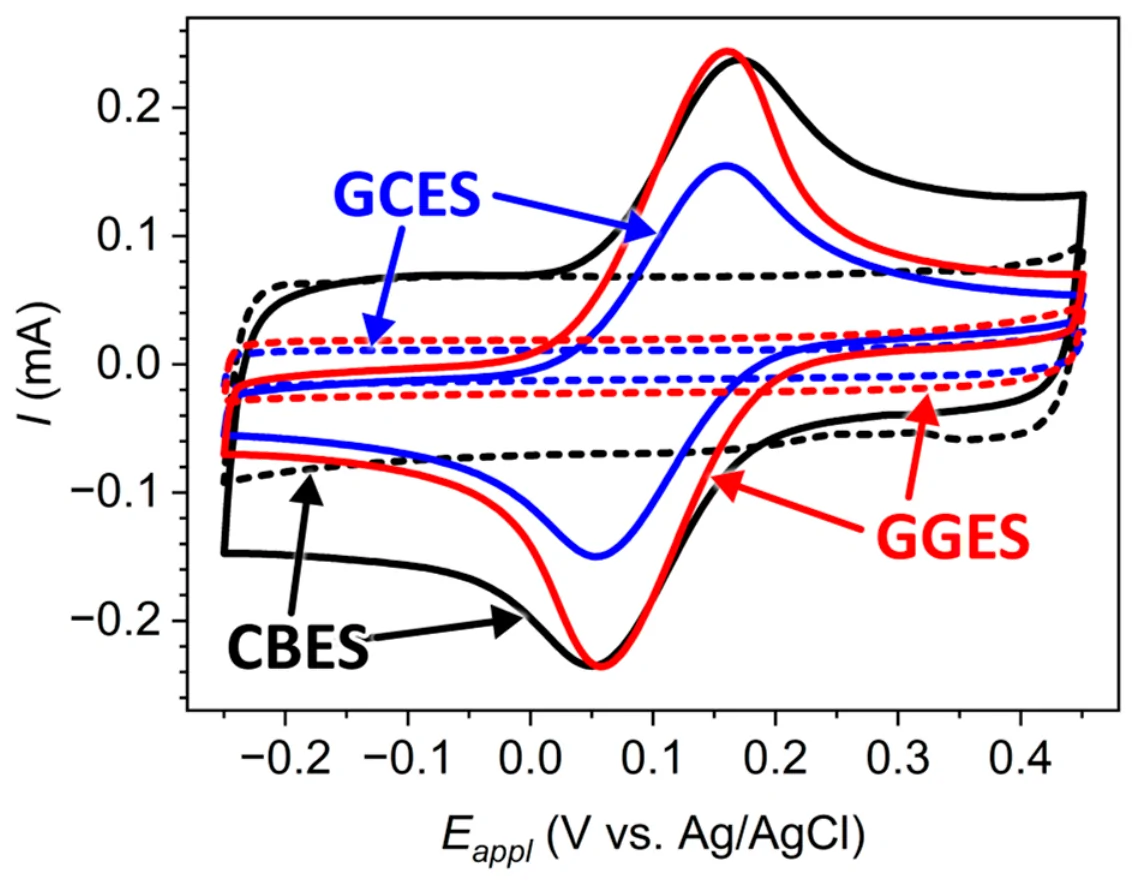
Cyclic voltammograms of IESs with graphite-glass (GGES), glassy carbon (GCES), and carbon black (CBES) WEs, recorded in 100 mM PBS (dashed) and 5 mM HCF (solid) at 100 mV s^−1^ between −0.25 V and +0.45 V, starting from an initial potential of −0.25 V in the positive direction.

The electrochemical behaviour of GGES, GCES, and CBES was investigated using the reversible [Fe(CN)_6_]^3−/4−^ (HCF) redox couple, a widely used surface-sensitive redox probe whose electron-transfer kinetics are strongly influenced by the WE material and its surface chemistry [7,16]. Because all IESs were fabricated with the same geometry and processing conditions and were evaluated using the same electrolyte, redox probe, and measurement conditions, the variation in their responses can be associated with the different WE material properties. The IESs were evaluated based on cyclic voltammograms (CVs) measured in 100 mM PBS and in a 5 mM HCF redox probe, with representative CVs shown in Figure 3. The CVs at different scan rates measured in PBS (Figure S4) were used to determine the double-layer capacitance (*C*_dl_), and those measured in HCF (Figure S5) were used to determine the electroactive surface area (*A*_ecsa_) and heterogeneous electron-transfer rate constants (*k*^0^). The uncompensated resistance (*R*_u_) values and parameters extracted from CVs are summarised in Table 2.

GGES, GCES, and CBES exhibit similar overall behaviour in the potential window between −0.25 V and +0.4 V, with featureless CVs in PBS and reversible redox peaks in HCF. The magnitude of the capacitive current (*I*_cap_) varies between electrodes, affecting how clearly the cathodic and anodic Faradaic current peaks (*I*_pc_, *I*_pa_) are distinguished from the capacitive background. GGES, GCES, and CBES demonstrate predominantly diffusion-controlled behaviour and good reversibility of the redox process, as indicated by *I*_pc_/*I*_pa_ ratios close to unity. The similar values of peak-to-peak separation (Δ*E*_p_) further confirm comparable electron-transfer behaviour across all IESs, regardless of the WE material. The nearly identical formal potentials (*E*_½_) indicate reproducible properties across all IESs. The observed differences in CVs arise from variations in the surface morphology, composition, and electrical properties of the WEs, consistent with a previous report [9]. The differences in *R*_u_ values, which are consistent with the WE resistivity data (Table 1), further confirm the influence of the electrical properties of the WEs on the electrochemical behaviour of the IESs.

GGES exhibits a moderate *C*_dl_, which can be attributed to the surface characteristics of the WE, such as surface roughness and the surface atomic O/C ratio [34]. The GG electrode possesses the highest surface roughness among the investigated WEs, with a low O/C ratio [11], resulting in a relatively hydrophobic surface that is expected to exhibit lower capacitance than hydrophilic surfaces [35]. It exhibits pronounced and well-defined redox peaks with a high peak-to-background (P/B) current ratio, indicating low background interference. *A*_ecsa_ is the highest among the tested IESs and scales with the macroscopic roughness of the WE. *k*^0^ is also the highest, consistent with the high intrinsic electrical conductivity of graphitic domains.

GCES exhibits the lowest *C*_dl_, as expected due to the similar hydrophobic nature of the GC surface, but with a smoother surface morphology than GG. It shows the most pronounced P/B ratio among the investigated IESs, indicating good signal definition. The *A*_ecsa_ is also lower than that of GGES but does not decrease proportionally with *C*_dl_, indicating that *A*_ecsa_ does not solely reflect the lower surface roughness of the WE. Instead, this suggests that a fraction of the surface accessible to the electrolyte is not diffusion-accessible and consequently electrochemically inactive towards HCF redox reactions. *k*^0^ is also lower, consistent with the lower conductivity and more disordered structure compared to G.

CBES displays the highest *C*_dl_, approximately three to five times greater than those of GGES and GCES, respectively. Although the CB has the smoothest surface at the micrometre scale, it is widely reported that its high capacitance originates from an extremely high specific surface area, nanoscale porosity, and a high density of structural defects [7]. The nanoscale structure and high degree of disorder in CB, as confirmed by STEM, result in greater surface oxide coverage, leading to a more hydrophilic surface. The large capacitive background leads to a substantially lower P/B current ratio, partially masking the Faradaic response. Nevertheless, the *A*_ecsa_ of CBES is comparable to that of GCES, with similar WE surface roughness. This indicates that a significant portion of the surface contributing to capacitance is not accessible for diffusion-controlled Faradaic processes. Despite higher electrical conductivity and presumably higher density of electronic states due to its more disordered structure of CB, the *k*^0^ of CBES is similar to that of GCES, suggesting that electron-transfer kinetics for the HCF redox probe are not strongly affected by these differences.

The electrochemical behaviour of GGES, GCES, and CBES closely follows the trends previously observed for the G (without added glass), GC, and CB square-shaped WEs, prepared from the same carbon materials under identical processing conditions, in the form of 8 mm × 8 mm squares, which were characterised in a conventional three-electrode configuration [9]. This observation confirms that integrating G, GC and CB into the IES configuration does not significantly alter the intrinsic properties of the carbon material. The geometric surface area (*A*_geo_) of the IES is 4.6 times smaller than that of the square-shaped WE, which is reflected in correspondingly lower *C*_dl_ and *A*_ecsa_ values. The measured *I*_cap_ for GCES and CBES are consistent with this trend, whereas GGES exhibits slightly higher values than expected. Since the smaller roughness of GG compared to G is not consistent with increased capacitance, this suggests that the addition of glass may contribute to surface charging. Overall, however, the incorporation of glass results in only a minor effect on the response of graphite-based electrodes. The *A*_ecsa_ values scale with the micrometre-scale surface roughness, and when normalised to *A*_geo_, the roughness factors (*A*_real_) are comparable to those of square-shaped WEs, indicating that observed differences primarily arise from intrinsic material properties. Notably, while GGES exhibits the highest accessible surface area, GCES and CBES show that a portion of the surface contributing to capacitance is not diffusion-accessible and not electrochemically active towards HCF redox reactions. In cases where the sensing application relies on diffusion-controlled reactions [9,36], *A*_ecsa_ provides a more relevant measure of the active surface accessible to the analytes than *C*_dl_. The peak current densities (*J*_p_) indicate lower values for GGES and similar values for GCES and CBES, which could be related to better electronic connectivity of the accessible electroactive surface within the film. The similar current density of GGES and a G square-shaped WE indicate that the addition of glass is likely not affecting the electrochemical response to HCF. The apparent *k*^0^ values are of the same order of magnitude (10^−3^ cm s^−1^) for all IESs, with small differences that correlate well with sheet resistance. Similar values for electrochemically pretreated IESs with graphite-glass WEs have been reported in our previous work [36]. The slightly higher *k*^0^ values compared to square-shaped WEs can be attributed to reduced ohmic drop due to shorter inter-electrode distances in the IES configuration.

These results demonstrate that, although electrode integration and geometry affect absolute values, the intrinsic electrochemical behaviour is governed primarily by the carbon WE material. The *A*_real_ and *k*^0^ values of all investigated IES are comparable to, or higher than, those reported for commercially available IESs [37]. Differences in capacitive behaviour can be directly correlated with the structural features revealed by STEM analysis. The highly ordered graphitic domains in GG promote rapid heterogeneous electron transfer. In contrast, the greater structural disorder in GC, and especially in CB, increase double-layer charging and surface wettability, while only a portion of the nanoscale surface remains electrochemically accessible to diffusion-controlled redox reactions.

An important observation of this study is that high double-layer capacitance should not be interpreted as direct evidence of a proportionally larger electrochemically active surface area for diffusion-controlled redox reactions. Although the CB electrodes exhibit the highest capacitance owing to their highly disordered nanoscale structure, indicating a high surface area, a substantial fraction of this surface is not readily accessible to the HCF redox probe. Consequently, capacitive and Faradaic electrochemical responses should be interpreted independently when evaluating carbon electrode materials. The observed differences in electrochemically accessible surface area and surface charging are expected to be key factors governing target analyte detection performance, especially where adsorption and accumulation effects contribute to the analytical response.

#### 3.3.2. Reproducibility, Operational Stability, and Shelf-Life

For practical sensing applications, IESs must provide not only suitable electrochemical characteristics but also reproducible fabrication and stable performance during operation and storage. Therefore, the reproducibility, operational stability, and shelf-life of the fabricated IESs were evaluated using the [Fe(CN)_6_]^3−/4−^ redox couple. Representative CVs for operational stability are shown in Figure S6, while Figure S7 shows open current potential (OCP) measurements, in which the stability of the Ag/AgCl-integrated RE was investigated. Representative CVs for shelf-life stability are shown in Figure S8.

The reproducibility of sensor fabrication was assessed from the *I*_pa_ and Δ*E*_p_ values obtained for three independently fabricated IESs. All IESs exhibited good reproducibility (Table 3). The RSD values of *I*_pa_ and Δ*E*_p_ below 3.5% demonstrate that the thick-film fabrication process yields highly reproducible electrochemical sensors, irrespective of the carbon material employed. The achieved fabrication reproducibility below 5% is well within the generally accepted performance criteria for analytical applications, as reported in the literature [38].

**Figure 4:**
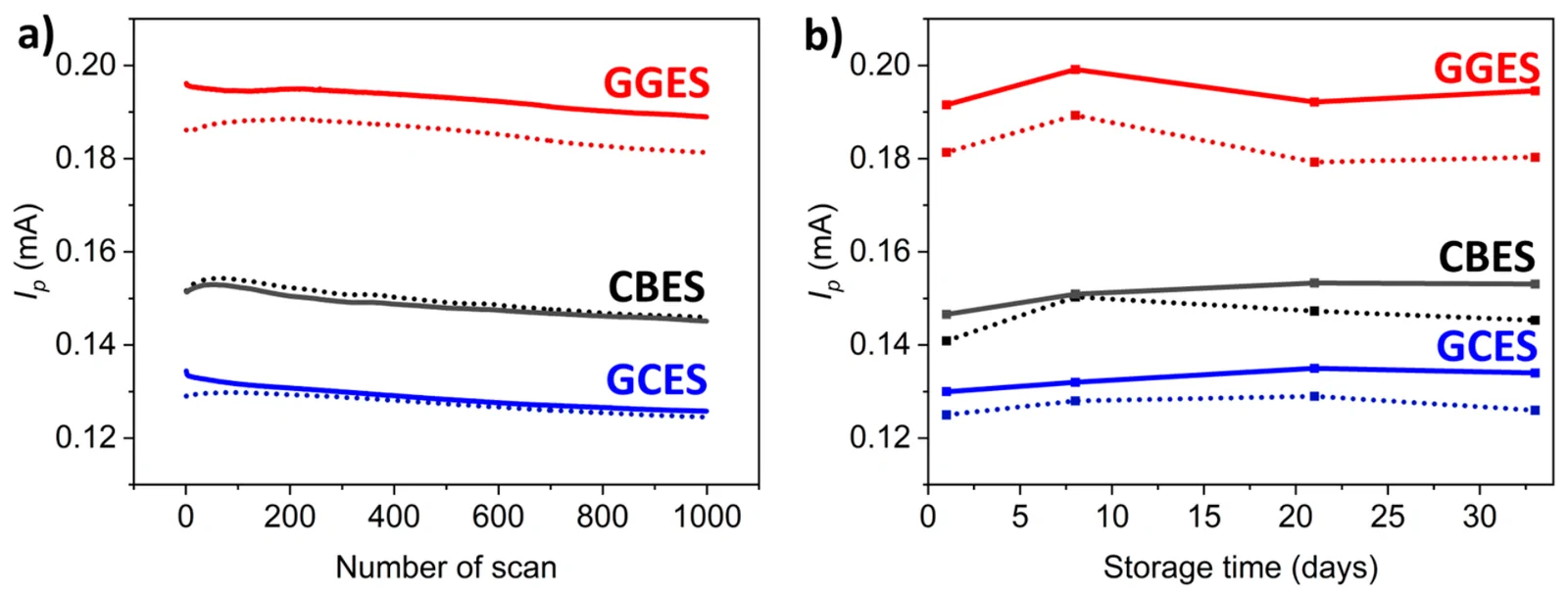
Dependence of absolute value of anodic (*I*_pa_, solid) and cathodic (*I*_pc_, dotted) peak currents (*I*_p_) on (**a**) number of consecutive scans and on (**b**) storage time for IESs with graphite-glass (GGES, red), glassy carbon (GCES, blue), and carbon black (CBES, black) WEs.

During 1000 consecutive CV cycles, all IESs exhibited similar behaviour (Figure S6). The peak currents gradually decreased with increasing numbers of scans, as shown in Figure 4. The final scan, however, retained more than 95% of the initial current response for all electrode materials. This small decrease in current is likely associated with gradual surface changes during prolonged electrochemical cycling rather than degradation of the electrode structure. The Δ*E*_p_ also remained essentially unchanged throughout the experiment, with RSD values below 1% (Figure S6). A slight negative drift of both anodic and cathodic peak potentials was observed, resulting in convergence of *E*_½_ from approximately 0.110 V to 0.090 V. The drift occurred predominantly during the first 100 cycles before stabilising. This shift is unlikely to originate from changes at the WE, as indicated by the open-circuit potential (OCP) measurement performed with the pseudo-RE as the WE against a conventional Ag/AgCl/3 M KCl RE (Figure S7). The OCP measurements showed a similar pseudo-RE potential drift of 0.020 V to that observed in *E*_½_, supporting the interpretation that the shift is primarily associated with the gradual equilibration of the pseudo-RE, while the WE remains electrochemically stable throughout cycling.

Storage under ambient laboratory conditions for up to 33 days had no significant effect on the electrochemical response of the IESs (Figure S8). No systematic changes in *I*_pa_ or Δ*E*_p_ were observed, with RSD values below 2% for all IESs. A small positive shift of approximately 10 mV in *E*_½_ became apparent after approximately 21 days of storage, which is most likely related to gradual oxidation of the pseudo-RE. Nevertheless, this shift had negligible influence on the voltammetric response, indicating good shelf-life stability of the fabricated sensors.

The results demonstrate that the developed fabrication procedure provides IESs with good reproducibility, operational durability, and satisfactory shelf-life irrespective of the carbon material used for the WE. The observed differences between graphite-, glassy carbon-, and carbon black-based electrodes therefore arise primarily from their intrinsic electrochemical characteristics rather than variability introduced during sensor fabrication.

#### 3.3.3. Operating Potential Window

The operating potential window (OPW) of the IESs was evaluated to confirm their suitability for the electroanalysis of various pollutants. Progressive scans are shown in Figure S9. Slight peaks appeared at each vertex potential but disappeared as the limits were extended to higher overpotentials, indicating electrochemical conditioning of the WE surface. In the literature, this behaviour in the cathodic region is attributed to the reduction of surface functionalities and removal of impurities in the WE material [11,22], whereas in the anodic region, the additional features likely arise from carbon oxidation, which leads to the formation of carbon dioxide or oxidised surface species [22,39]. The presence of additional surface processes is further supported by the increase in the apparent capacitive current observed in CVs recorded over an extended potential range. A gradual shift of the apparent onset potentials of the HER and oxygen evolution reaction (OER) towards higher overpotentials was also observed during progressive cathodic and anodic polarisation, respectively, across all IESs. The current response stabilised with repeated cycling at constant vertex potentials, indicating decreased HER and OER activity upon conditioning and thus extended applicable potential windows.

**Figure 5:**
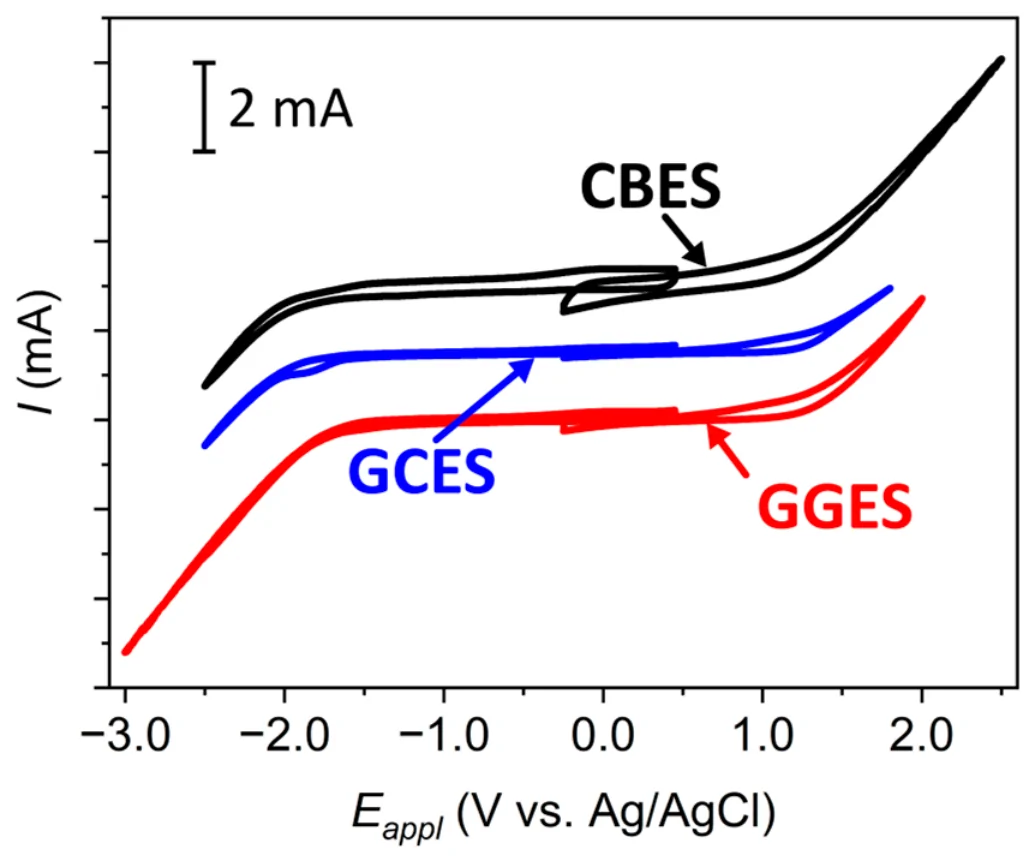
Electrochemical activity of IESs with graphite-glass (GGES, red), glassy carbon (GCES, blue), and carbon black (CBES, black) WEs in 0.1 M PBS in the widest potential range without delamination of the WE.

The OPW limits were identified as −1.55 V to +0.98 V for GGES, consistent with a previous report [11], −1.63 V to +0.92 V for GCES, and −1.41 V to +0.70 V for CBES. Although the limits of the OPW were investigated between −2.0 V and +1.5 V, higher potentials were tested by changing the vertex potentials beyond this range by 500 mV increments until delamination of the WE from the substrate occurred. The CVs measured over the widest cathodic and anodic potential range without delamination are shown in Figure 5. All IESs can withstand overpotentials of at least −2.5 V and +1.5 V, with GGES demonstrating superior stability in the negative potential range and CBES in the positive potential range. All IESs exhibit reproducible and stable electrochemical behaviour with a wide OPW, which is promising for sensing applications.

## 4. Conclusions

Integrated electrochemical sensors with Pt counter electrode and electrical contacts, an Ag/AgCl reference electrode, and three carbon-based working electrodes, namely graphite-glass (GG), glassy carbon (GC), and carbon black (CB), were successfully fabricated by screen printing. Structural characterisation of the working electrodes, annealed at 850 °C in argon, by electron microscopy and diffraction demonstrated that the GG thick film exhibited highly ordered sp^2^-hybridised carbon, along with less ordered amorphous regions and a glassy phase. The GC thick film consists predominantly of a disordered structure with local graphitic ordering. The CB thick film consists of highly disordered carbon with localised structural ordering within carbon nanoparticles composed of curved graphitic shells. These structural and morphological differences were reflected in their electrochemical properties, demonstrating that the selection of the carbon working-electrode material provides an effective means of tuning sensor performance. The developed sensors exhibited highly reproducible electrochemical behaviour, with a relative standard deviation of *I*_pa_ and Δ*E*_p_ below 3.5% and a broad operating potential window from −1.6 V to +1.0 V. The sensors maintain stable electrochemical responses over 1000 cyclic voltammetry cycles, with a decrease in peak current of less than 5%, and shelf stability of 33 days with a relative standard deviation below 2%. These results demonstrate that the developed IESs combine reproducible electrochemical performance, operational durability, and storage stability, which are important requirements for practical sensing applications.

The screen-printing approach provides a scalable and cost-effective route to integrated electrochemical sensors, while the distinct structural characteristics of GG, GC, and CB offer flexibility for tailoring them to specific analytical requirements. Future work should therefore focus on exploiting this relationship between structure and properties to optimise the electrode material for specific target analytes and on validating sensor performance in real samples and application-relevant conditions. Such studies could support the translation of the developed platform from laboratory-scale electrochemical characterisation towards practical, application-specific sensing devices.

## Figures and Tables

**Table 1:** Average thickness, roughness, sheet resistance, and resistivity of GG, GC, CB, Pt, and Ag/AgCl thick-film electrodes.

Electrode	*t* [μm]	*R*_q_ [μm]	*R*_s_ [Ω/sq]	*ρ* [mΩ cm]
GG	36.1 ± 3.2	2.6 ± 0.2	8.7 ± 0.6	31
GC	15.4 ± 0.2	0.7 ± 0.1	39.2 ± 2.4	60
CB	16.5 ± 0.4	0.4 ± 0.1	30.4 ± 2.8	50
Pt	8.5 ± 0.6	0.7 ± 0.1	(4.8 ± 0.2) × 10^−3^	0.004
Ag/AgCl	17.7 ± 2.8	3.8 ± 0.9	(50.7 ± 0.3) × 10^−3^	0.09

**Table 2:** Electrochemical properties of IESs with graphite-glass (GGES), glassy carbon (GCES), and carbon black (CBES) WEs obtained from CVs recorded in PBS and HCF.

Parameter	GGES	GCES	CBES
*R*_u_ [Ω]	140 ± 2	167 ± 3	146 ± 1
*I*_cap_ [μA]	42 ± 4	21 ± 1	141 ± 2
*C*_dl_ [μF]	508 ± 35	299 ± 7	1458 ± 44
P/B current ratio	10.2 ± 0.7	13.5 ± 0.4	2.3 ± 0.1
−*I*_pc_ [mA]	0.213 ± 0.003	0.140 ± 0.002	0.159 ± 0.003
*I*_pa_ [mA]	0.224 ± 0.004	0.144 ± 0.001	0.166 ± 0.004
*J*_p_ [mA cm^−2^]	0.36 ± 0.04	0.45 ± 0.04	0.42 ± 0.04
*I*_pc_/*I*_pa_	0.95 ± 0.01	0.97 ± 0.01	0.96 ± 0.01
Δ*E*_p_ [mV]	102.5 ± 1.4	105.0 ± 3.7	119.6 ± 1.4
*E*_½_ [mV]	114 ± 3	110 ± 4	113 ± 2
*A*_ecsa_ [cm^2^]	0.61 ± 0.05	0.31 ± 0.02	0.39 ± 0.02
*A*_real_ [cm^2^/cm_geo_^2^]	4.36 ± 0.39	2.24 ± 0.15	2.79 ± 0.18
*k*^0^ [10^−3^ cm/s]	15.47 ± 0.42	8.05 ± 0.50	7.64 ± 0.16

**Table 3:** Summary of the electrochemical properties (*I*_pa_ and Δ*E*_p_) of the GGES, GCES, and CBES obtained from the CVs recorded during reproducibility, operational stability, and shelf-life evaluation in HCF.

Electrode	Reproducibility		Operational Stability		Shelf-Life Stability	
	RSD-*I*_pa_ (%) (N = 3)	RSD-Δ*E*_p_ (%) (N = 3)	*I*_pa,1000_/*I*_pa,1_	RSD-Δ*E*_p_ (%)	RSD-*I*_pa_ (%) (N = 4)	RSD-Δ*E*_p_ (%) (N = 4)
GGES	1.8	1.4	96.2	0.9	1.7	1.5
GCES	0.7	3.5	95.9	0.8	1.7	1.5
CBES	2.4	1.2	95.2	0.9	1.9	1.6

## Data Availability

The data that support the findings of this study are available from the Zenodo repository [40].

## Supplementary Materials

The following supporting information can be downloaded at: https://www.mdpi.com/article/10.3390/s26165260/s1, Figure S1: BF-STEM image of GG thick film with corresponding EDXS analysis; Figure S2: EELS spectra of GG, GC, and CB thick films; Figure S3: Thickness profiles of GGES, GCES, and CBES with Pt CE, Ag/AgCl RE, and either GG, GC, or CB WE on alumina substrate; Figure S4: Cyclic voltammograms recorded in 0.1 M PBS supporting electrolyte at different scan rates using GGES, GCES, and CBES with dependence of *I*_cap_; Figure S5: Cyclic voltammograms recorded in 5 mM HCF in 0.1 M PBS supporting electrolyte at different scan rates using GGES, GCES, and CBES with dependence of *I*_pa_ on *ν*^1/2^ and dependence of *Ψ* on *ν*^−1/2^; Figure S6: Selected cyclic voltammograms of operational stability measurements; Figure S7: Open current potential measured between Ag/AgCl pseudo-RE and a conventional Ag/AgCl/3 M KCl RE; Figure S8: Selected cyclic voltammograms of shelf-life stability measurements; Figure S9: Progressive CV scans measured in 0.1 M PBS supporting electrolyte using GGES, GCES, and CBES, used for the determination of the operating potential window (OPW).

## Abbreviations

The following abbreviations are used in this manuscript:

IES	Integrated electrochemical sensor
SPE	Screen printed electrodes
WE	Working electrode
G	Graphite
GC	Glassy carbon
CB	Carbon black
CE	Counter electrode
RE	Reference electrode
GG	Graphite-glass
XRD	X-ray diffraction
GI	Grazing incidence
(S)TEM	(Scanning) transmission electron microscopy
SAED	Selected area diffraction
EDXS	Energy-dispersive X-ray spectroscopy
EELS	Electron energy loss spectroscopy
PIPS	Precision ion polishing system
PBS	Phosphate buffer solution
HCF	Hexacyanoferrate (II)/(III)
CV	Cyclic voltammetry
GGES	Integrated electrochemical sensor with graphite-glass working electrode
GCES	Integrated electrochemical sensor with glassy carbon working electrode
CBES	Integrated electrochemical sensor with carbon black working electrode
RSD	Relative standard deviation
OCP	Open current potential
OPW	Operating potential window
HER	Hydrogen evolution reaction
AO	Aluminium oxide
DF	Dark-field
XPS	X-ray photoelectron spectroscopy
OER	Oxygen evolution reaction
