# Supplementary material for: Electrochemical Sensors with Carbon-Based Thick-Film Working Electrodes: Correlating Structure with Electrochemical Performance, Reproducibility, and Stability
Source: Sensors (Basel). 2026 Aug 19;26(16):5260. doi: 10.3390/s26165260 (PMC13517454; doi:10.3390/s26165260)
Supplement: Supplementary file 1 [file sensors-26-05260-s001.zip › sensors-4489339-supplementary.pdf]

Supplementary Information

Electrochemical Sensors with Carbon-Based Thick-Film Working Electrodes: Correlating Structure with Electrochemical Performance, Reproducibility, and Stability

Barbara Repič [1,2], Gregor Marolt [3], Andreja Benčan Golob [1], Goran Dražić [1] and Danjela Kuscer [1,2,*]

In this work, integrated electrochemical sensors (IESs) composed of a Pt counter electrode (CE), an Ag/AgCl pseudo-reference electrode (RE), and a carbon-based working electrode (WE), integrated onto an alumina substrate, were investigated. WEs were prepared from powders of three different carbon allotropes, namely graphite (G), glassy carbon (GC), and carbon black (CB). While adhesion of pure GC and CB thick films to alumina was sufficient, a mixture of graphite and glass (GG) in a 75/25 weight ratio was used for GG thick films due to improved adhesion to alumina.

Figure S1 shows a bright-field scanning transmission electron microscopy (BF-STEM) image of the GG thick film, together with corresponding energy-dispersive X-ray spectroscopy (EDXS) elemental maps for C, Si, Al, Ca, and O. These results confirm the presence of two distinct phases within the film. The irregular, micrometre-sized darker particles correspond to the glass phase, while the brighter regions with a layered morphology correspond to graphite, as discussed in the manuscript. Since an alkaline silicate glass powder was used for film preparation, Ca was detected in the glass phase in addition to SiO_2.

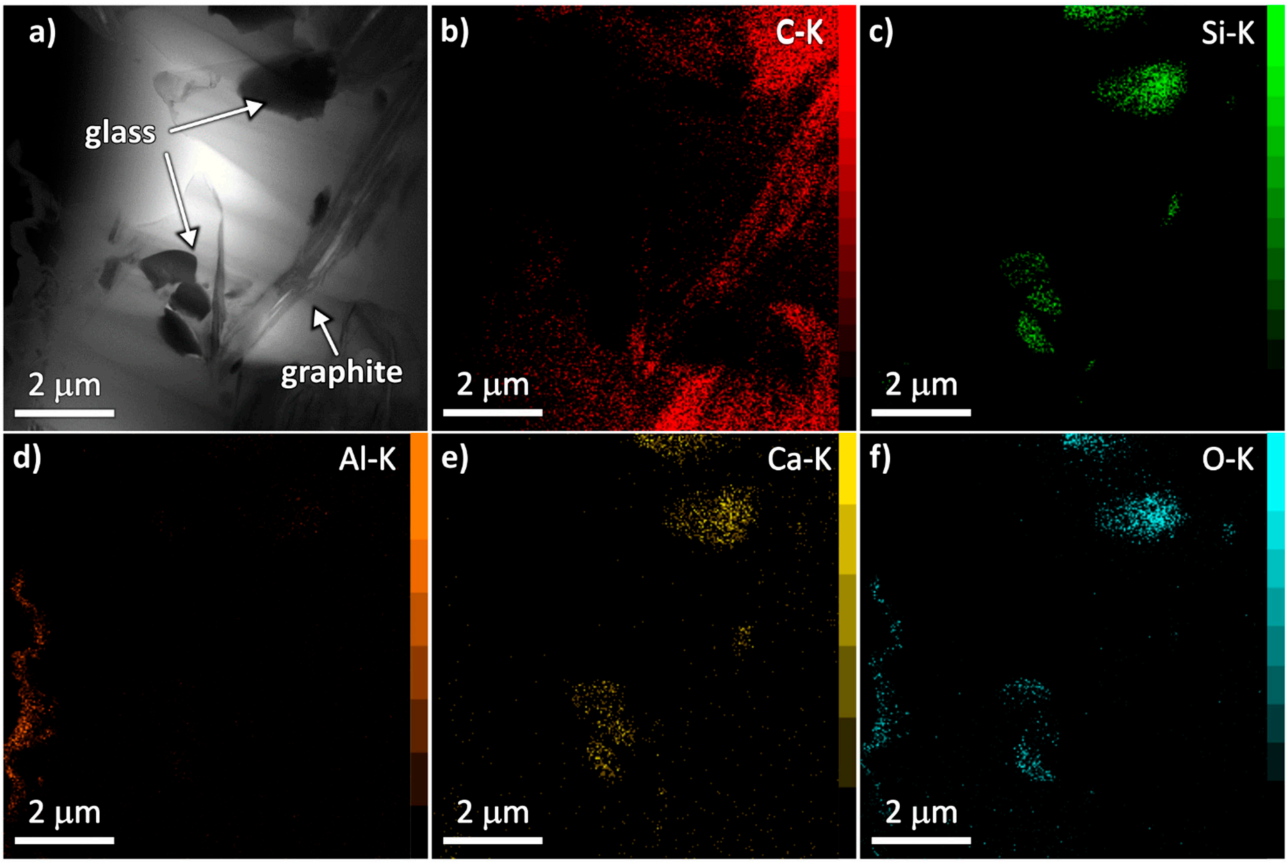

Figure S1: BF-STEM image of GG thick film (a), with corresponding EDXS analysis for K-shell emissions of: b) carbon (C-K), c) silicon (Si-K), d) aluminium (Al-K), e) calcium (Ca-K), and f) oxygen (O-K).

Figure S2 shows electron energy loss spectroscopy (EELS) spectra of GG, GC, and CB thick films, and a dark-field scanning transmission electron microscopy (DF-STEM) image of GG thick film. The elongated layered structures exhibit a different spectrum from the adjacent region, which are marked in the DF-STEM image as A and B, respectively. All three carbon thick films display the π* peak around 285 eV and a broader σ* peak around 292 eV, but with different ratios of the π* to σ* peaks, indicating different fractions of sp^2-hybridised carbon atoms in the material, as discussed in the manuscript.

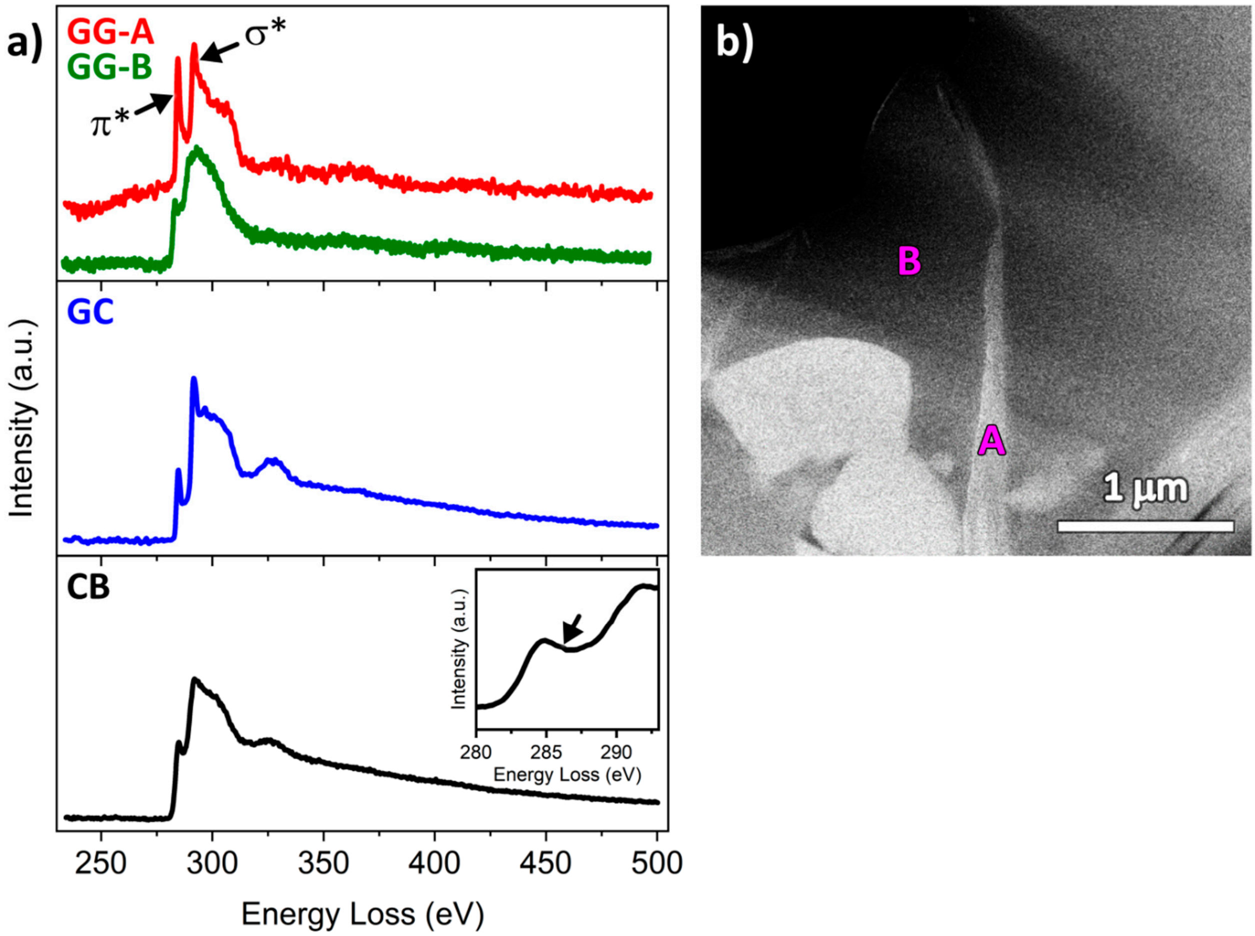

Figure S2: a) EELS spectra of GG, GC, and CB thick films, and b) DF-STEM image of GG thick film showing brighter elongated layered structures (A) and the darker adjacent region (B), which exhibited spectra GG-A and GG-B, respectively.

Figure S3 shows representative thickness profiles of IESs with a Pt CE, Ag/AgCl RE, and carbon-based working electrode (WE) integrated onto an alumina substrate. From the profiles of IESs with GG WE (GGES), GC WE (GCES), and CB WE (CBES), it is evident that all thick-film electrodes exhibit a uniform thickness across the entire cross-section of the film, and their thickness and roughness are summarised in the manuscript.

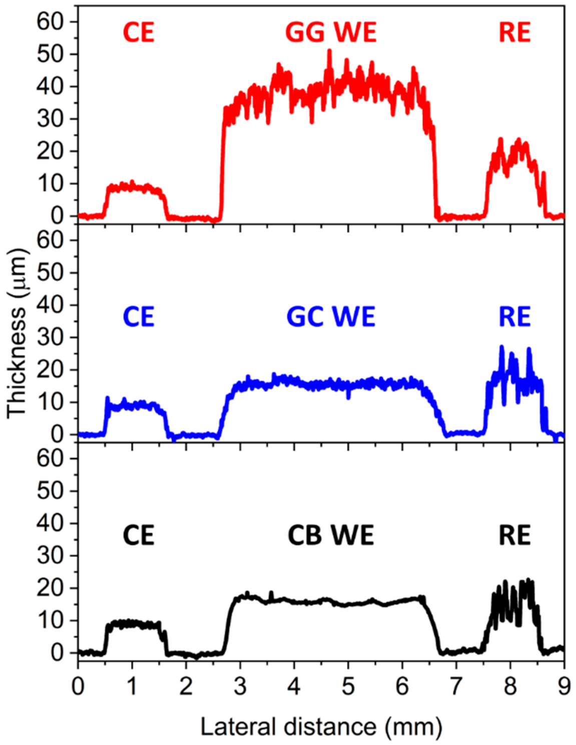

Figure S3: Thickness profiles of GGES, GCES, and CBES with Pt CE, Ag/AgCl RE, and either GG, GC, or CB WE on an alumina substrate.

Figure S4a–c shows cyclic voltammograms (CVs) measured using GGES, GCES, and CBES in 0.1 M phosphate buffer solution (PBS) as supporting electrolyte at scan rates (ν) of 50, 75, 100, 150, and 200 mV s^{-1}. With increasing ν, the capacitive current (I_{cap}) increases linearly for all IES responses. Figure S4d shows the dependence of I_{cap} on ν, and the slopes of these relations were used to determine the double-layer capacitance (C_{dl}) reported in Table 1 of the manuscript.

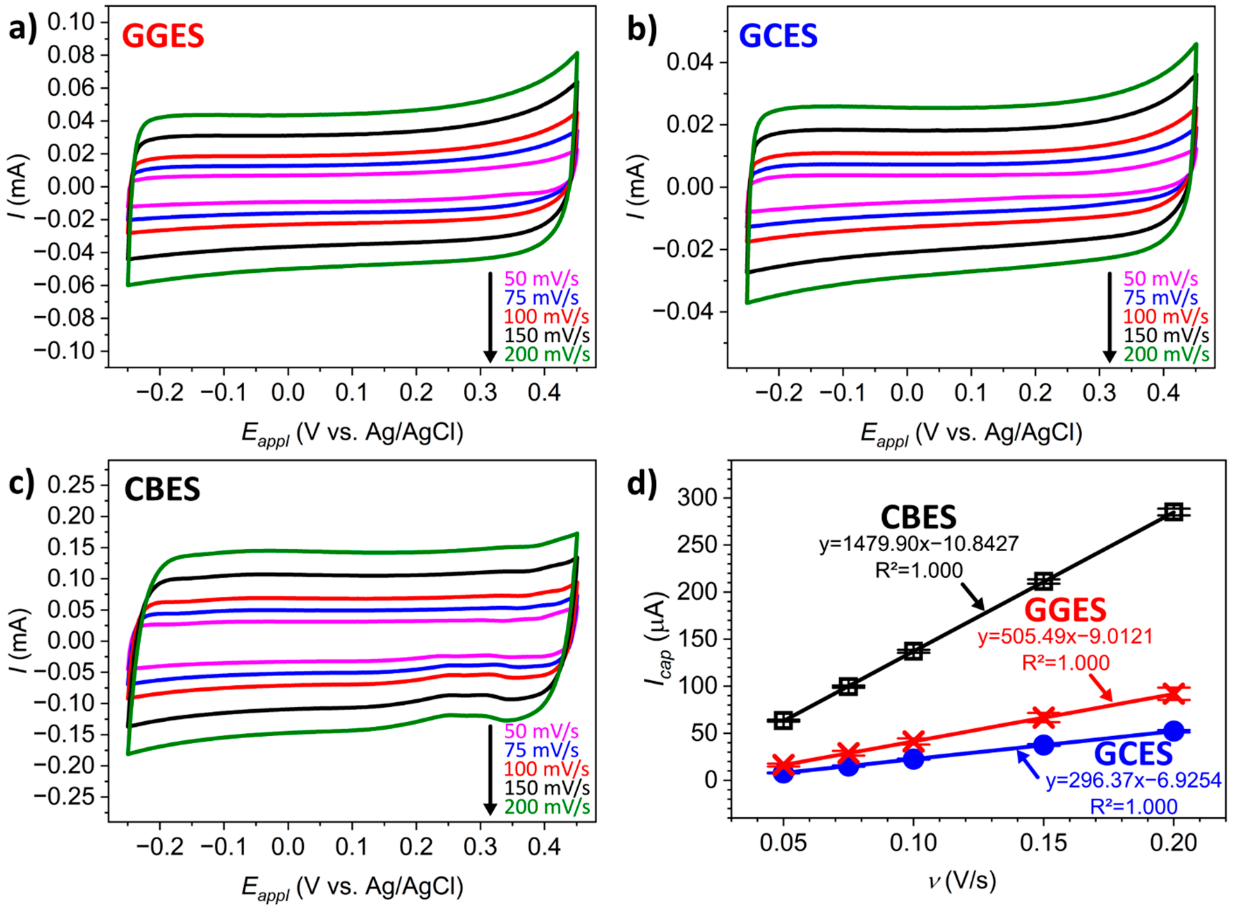

Figure S4: Cyclic voltammograms recorded in 0.1 M PBS supporting electrolyte at scan rates of 50, 75, 100, 150, and 200 mV s^{-1} between −0.25 V and +0.45 V, starting from an initial potential of −0.25 V in the positive direction, using a) GGES, b) GCES, and c) CBES. d) Dependence of I_{cap} on ν for GGES (red crosses), GCES (blue circles), and CBES (black squares).

Figure S5a–c shows CVs measured on GGES, GCES, and CBES in a 5 mM $[Fe(CN)_6]^{3-/4-}$ (HCF) redox probe in 0.1 M phosphate buffer solution (PBS) as the supporting electrolyte at scan rates (ν) of 50, 75, 100, 150, and 500 mV s^{-1}. With increasing ν, both the anodic (I_{pa}) and cathodic peak currents (I_{pc}) increase, and the anodic (E_{pa}) and cathodic peak potentials (E_{pc}) shift outwards for all IES responses. The dependence of the peak potentials on ν indicates that this redox couple exhibits quasi-reversible behaviour on the prepared IESs. Figure S5d shows the dependence of I_{pa} on the square root of the scan rate ($\nu^{1/2}$). Linear dependence on $\nu^{1/2}$ (rather than on ν) indicates that the electrochemical process can be characterised as diffusion-controlled, and the slopes of these relations were used to determine the electrochemically active surface areas (A_{ecsa}) reported in Table 1 of the manuscript. Furthermore, the slopes of the linear dependence of log $|I_{pa}|$ on log $|\nu|$ of 0.61 (R^2 = 0.9988), 0.52 (R^2 = 0.9994), and 0.48 (R^2 = 0.9937) for GGES, GCES, and CBES, respectively, indicate that the redox reaction of HCF is in fact mainly diffusion-controlled for GCES and CBES, whereas a mixed control regime with a non-negligible contribution from adsorption is suggested for GGES. Figure S5e shows the dependence of Nicholson's dimensionless parameter (Ψ) on the inverse of the square root of the scan rate ($\nu^{-1/2}$). The slopes were used to determine the heterogeneous electron transfer rate constants (k^0) reported in Table 1 of the manuscript.

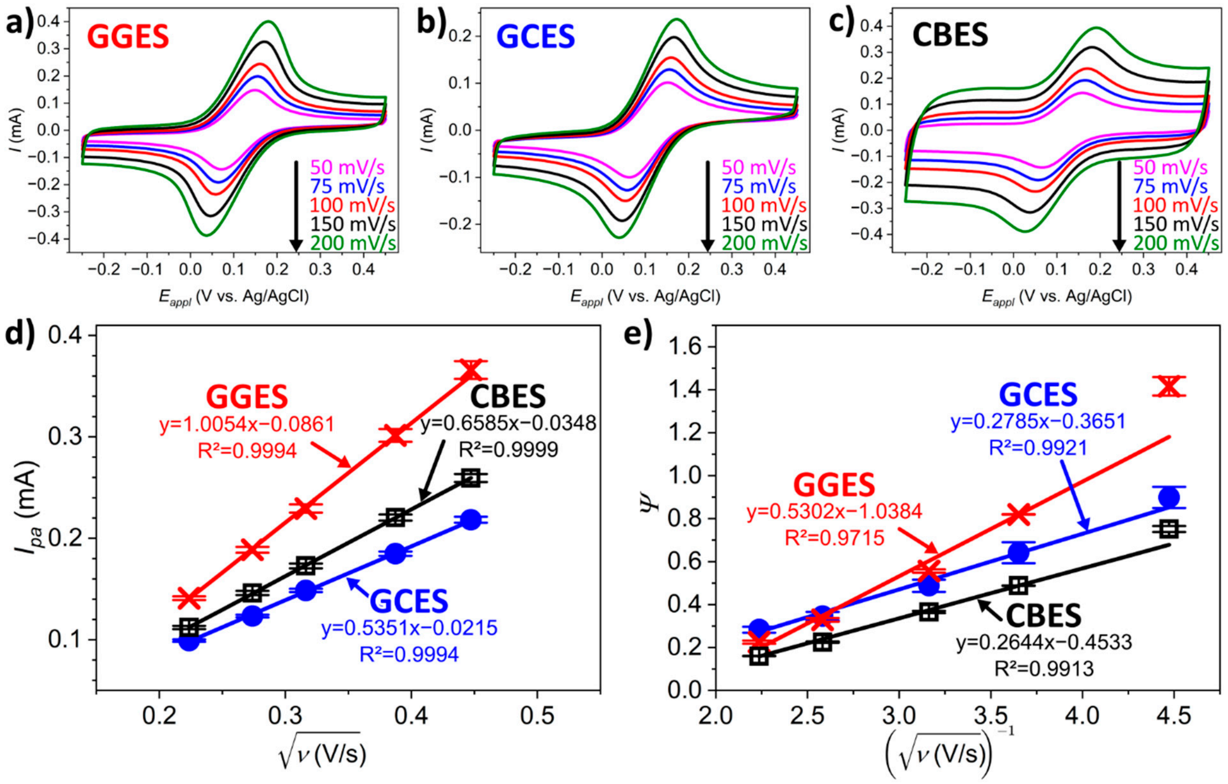

Figure S5: Cyclic voltammograms recorded in 5 mM HCF in 0.1 M PBS supporting electrolyte at scan rates of 50, 100, 200, and 500 mV s^{-1} between −0.25 V and +0.45 V, starting from an initial potential of −0.25 V in the positive direction, using a) GGES, b) GCES, and c) CBES. d) Dependence of I_{pa} from the first anodic scan on $\nu^{1/2}$ and e) dependence of Ψ on $\nu^{-1/2}$ for GGES (red crosses), GCES (blue circles), and CBES (black squares).

Figure S6a–c shows selected CVs recorded using GGES, GCES, and CBES in 5 mM HCF in 0.1 M PBS at 100 mV s^{-1} during the measurement of 1000 consecutive scans to evaluate operational stability. Similar trends are observed on all IESs regardless of the WE material. The peak currents gradually decrease and after 1000 cycles the I_{pa} falls to 96.2 %, 95.9 %, and 95.2 % of the initial value for GGES, GCES, and CBES, respectively. ΔE_P, however, remains stable throughout all 1000 scans, with RSD values of 0.9 %, 0.8 %, and 0.9 % for GGES, GCES, and CBES, respectively. Since IESs have a pseudo-RE, which is less stable than conventional bulk electrodes, some negative drift is observed in both anodic and cathodic peaks. The $E_{½}$ value drifts towards 0.090 V for all IESs. The majority of this drift occurs within the first 100 cycles, after which it is relatively stable.

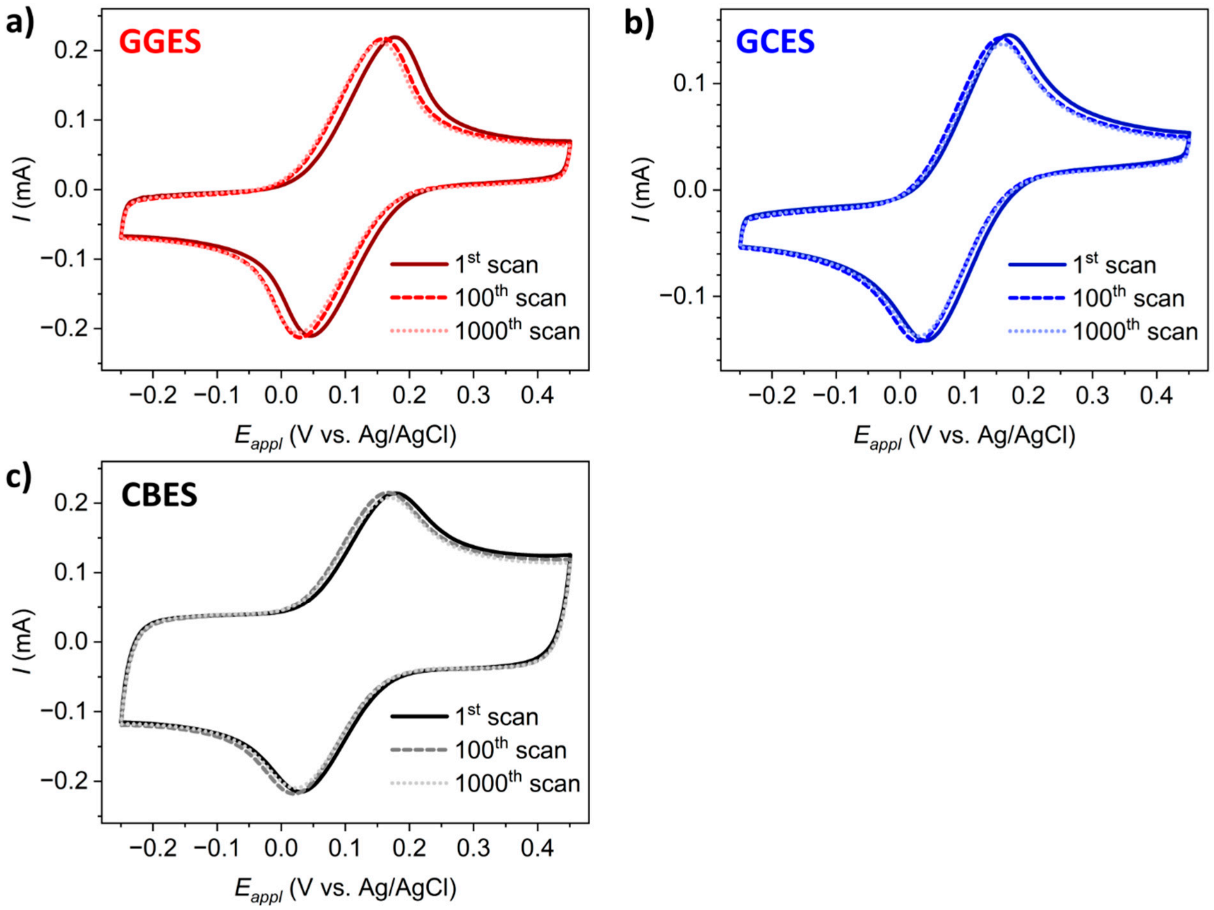

Figure S6: Cyclic voltammograms of the 1st (solid), 100th (dashed) and 1000th (dotted) scan recorded in 5 mM HCF in 0.1 M PBS supporting electrolyte at 100 mV s^{-1} between −0.25 V and +0.45 V, starting from an initial potential of −0.25 V in the positive direction, during measurement of 1000 consecutive scans using a) GGES, b) GCES, and c) CBES.

Figure S7 shows open current potential measurement between two parallels of Ag/AgCl pseudo-RE integrated onto IES connected as the WE against a conventional Ag/AgCl/3 M KCl RE for the duration of 1 hour. The results show an almost linear drift for approximately 20 mV toward more positive potentials, confirming the pseudo-RE potential changes with time. As described in the manuscript, the formal potential ($E_{½}$) drifts during operational stability study from approximately 0.110 V towards 0.090 V, where it stabilises. The similar drift of 20 mV further confirms the equilibration of the RE integrated onto IES causes the peak potential drifts observed in CVs with consecutive measurement of 1000 cycles.

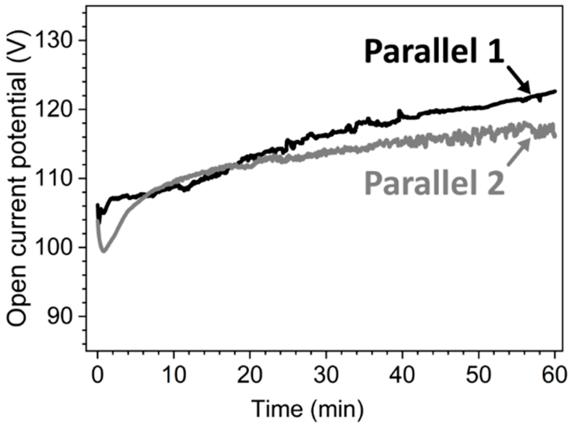

Figure S7: Open current potential measured in 5 mM HCF in 0.1 M PBS-supporting electrolyte between Ag/AgCl pseudo-RE and a conventional Ag/AgCl/3 M KCl RE for the duration of 1 hour.

Figure S8a–c shows selected CVs recorded on GGES, GCES, and CBES in 5 mM HCF in 0.1 M PBS at 100 mV s⁻¹ during long-term stability evaluation. Independently prepared IESs were stored under ambient laboratory conditions for 0, 8, 21, and 33 days prior to electrochemical characterisation in HCF. Similar trends are observed on all IESs regardless of the WE material. The peak currents show no specific trends, and the RSD of I_{pa} was 1.7 %, 1.7 %, and 1.9 % for GGES, GCES, and CBES, respectively. The ΔE_p is also stable during storage, with RSD values of 1.5 %, 1.5 %, and 1.6 % for GGES, GCES, and CBES, respectively. The $E_{½}$ value, on the other hand, shows a positive drift of approximately +0.010 V after 21 days of storage. This drift is most likely associated with oxidation of the pseudo-RE.

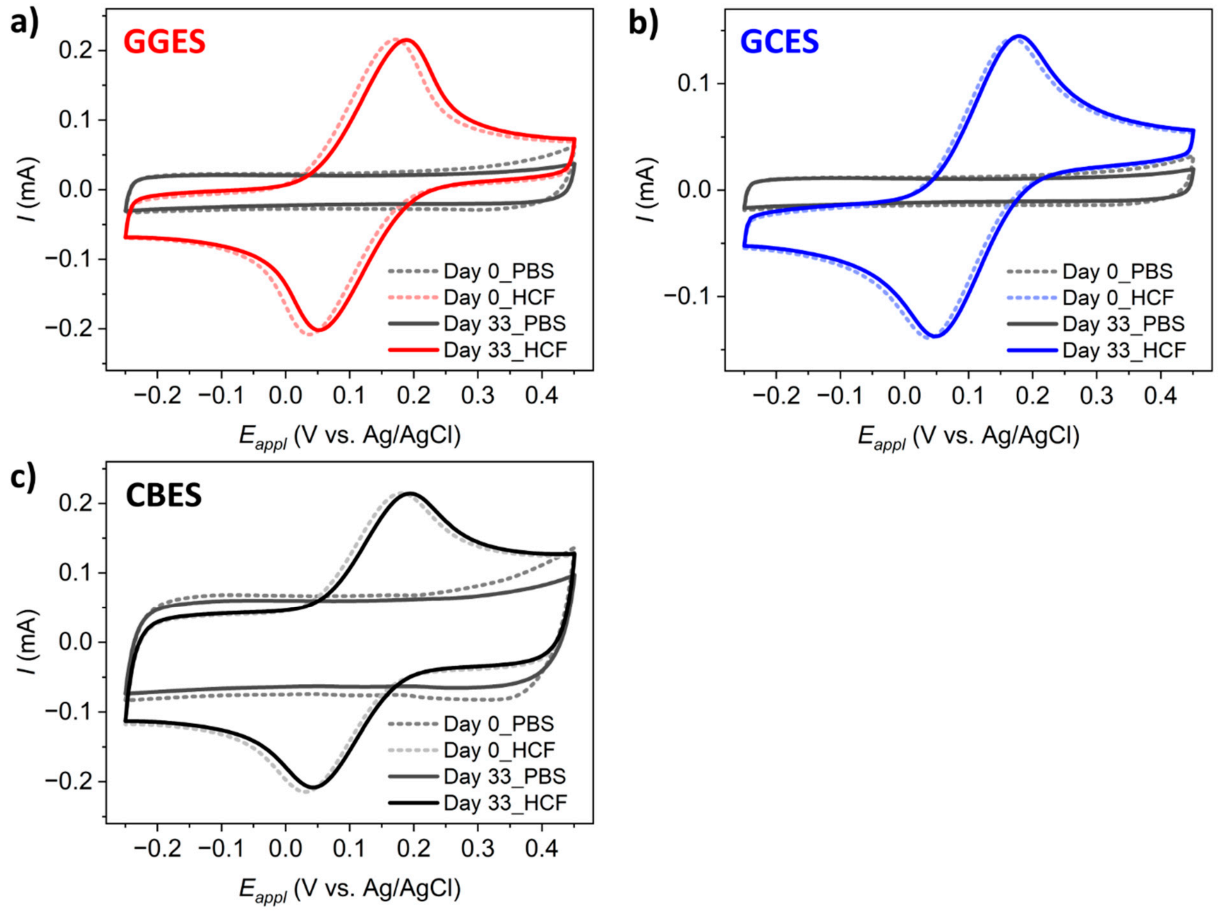

Figure S8: Cyclic voltammograms of a fresh (dashed) and 33-day-old (solid) IES recorded in 5 mM HCF in 0.1 M PBS supporting electrolyte at 100 mV s⁻¹ between −0.25 V and +0.45 V, starting from an initial potential of −0.25 V in the positive direction, during long-term stability measurements using: a) GGES, b) GCES, and c) CBES.

Figure S9a–c shows a series of CVs measured on GGES, GCES, and CBES in 0.1 M PBS, where the lower and upper vertex potentials were incrementally changed for 50 mV increments from initial vertex potential values of −0.25 V and +0.45 V until −2.0 V was reached during cathodic polarisation and +1.5 V during anodic polarisation. A separate IES was used for the progressive scan measurements in each potential range. A gradual shift of the apparent onset potentials of the hydrogen evolution reaction (HER) and oxygen evolution reaction (OER) towards higher overpotentials was observed for all IESs. The limits of the operating potential window (OPW) of the IESs reported in the manuscript were identified as the onset potentials of HER and OER in the final scans of each potential range, at which the current becomes equal to the peak current of the HCF redox probe, as shown in Figure S9a–c.

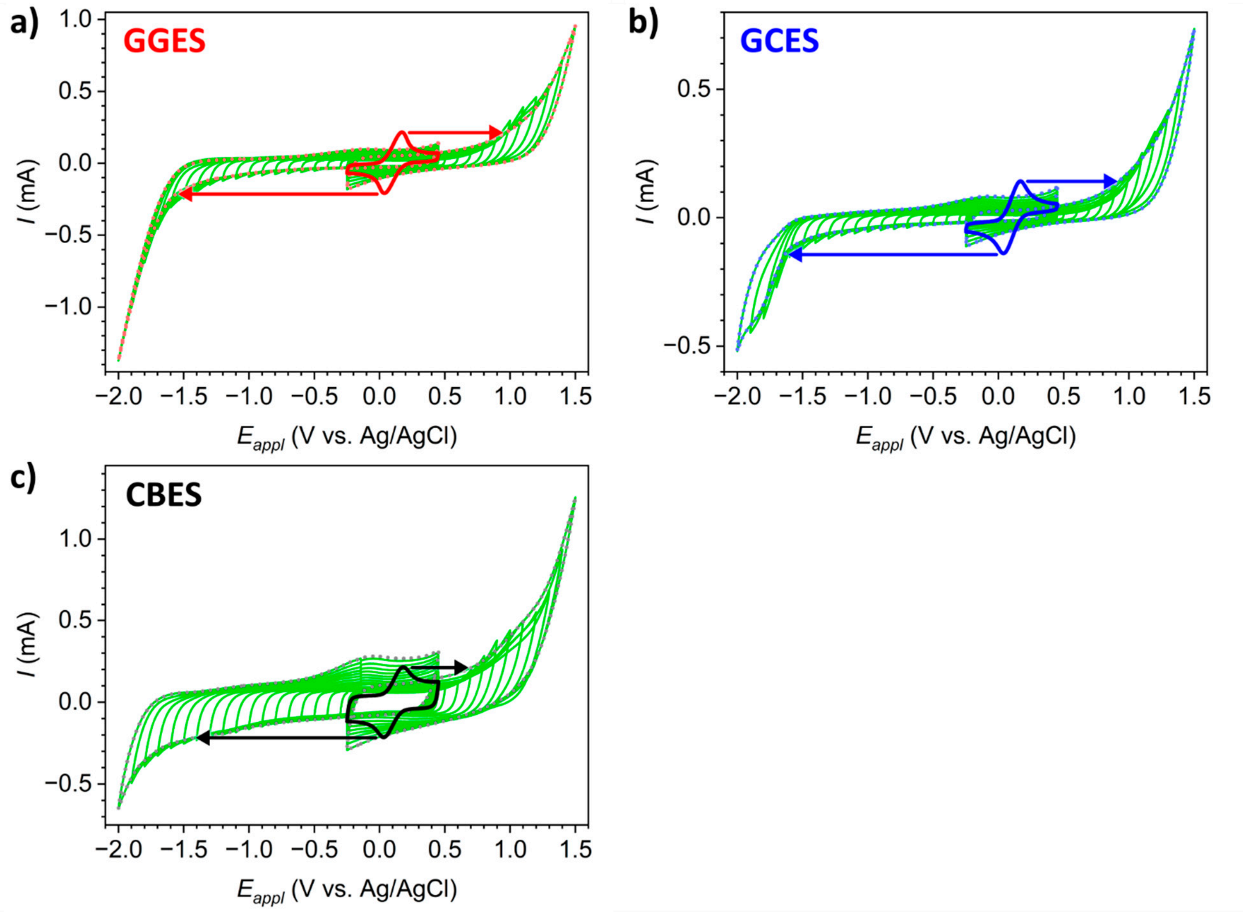

Figure S9: Progressive CV scans measured in 0.1 M PBS supporting electrolyte at a scan rate of 100 mV s^{-1}, with the vertex potentials of the sweep range changed in 100 mV increments from −0.25 V to −2.0 V in the cathodic and from +0.45 V to +1.5 V in the anodic potential range (green solid lines), with final scans highlighted with bright dotted lines, together with representative CVs recorded in 5 mM HCF in 0.1 M PBS-supporting electrolyte (dark solid lines) at 100 mV s^{-1} between −0.25 V and +0.45 V using a) GGES, b) GCES, and c) CBES for the determination of OPW.
